# Supplementary material for: Changes in Loneliness, BDNF, and Biological Aging Predict Trajectories in a Blood-Based Epigenetic Measure of Cortical Aging: A Study of Older Black Americans
Source: Genes (Basel). 2023 Mar 31;14(4):842. doi: 10.3390/genes14040842 (PMC10138024; doi:10.3390/genes14040842)
Supplement: Supplementary file 1 [file genes-14-00842-s001.zip › genes-2249918-supplementary.pdf]

Online Supplement Table S1

Partial Correlations between Cortical Age-Bd and MoCA subscales controlling for epigenetic clocks

	Zero correlation	Controlling Pace	Controlling GrimAge	Controlling PhenoAge
Visual Spatial	-.258**	-.260**	-.259**	-.203**
Attention Tap	-.203**	-.196**	-.132†	-.183*
Language fluency	-.227**	-.222**	-.153*	-.195**
Abstraction total	-.197**	-.175*	-.088	-.067
Delayed Recall	-.126†	-.119	-.017	-.169*
Orientation Total	-.216**	-.226**	-.115	-.168*
MoCA	-.278**	-.270**	-.152*	-.216**

† $p < 0.1$, * $p < 0.05$, ** $p < 0.01$ (two-tailed tests).

Supplemental Table S2

The list of the CpG sites in BDNFm

CpG index for 1stExon	CpG index for Promoter 1
cg09505801	cg07704699
cg21291635	cg10635145
cg06025631	cg12448003
cg07159484	cg04106006
cg22043168	cg06684850
cg27193031	cg01225698
cg26949694	cg11718030
cg25412831	cg15462887
cg06816235	cg10022526
cg24650785	cg25156688
cg11241206	cg06046431
cg04672351	cg01583131
cg20340655	cg24249411
cg09606766	
cg27351358	
cg02527472	
cg01642653	
cg16257091	
